# An Asynchronous Real-Time Corner Extraction and Tracking Algorithm for Event Camera

**DOI:** 10.3390/s21041475

**Published:** 2021-02-20

**Authors:** Jingyun Duo, Long Zhao

**Affiliations:** School of Automation Science and Electrical Engineering, Beihang University, Beijing 100191, China; JYDuo@buaa.edu.cn

**Keywords:** asynchronous, corner event, corner extraction, corner tracking, event camera

## Abstract

Event cameras have many advantages over conventional frame-based cameras, such as high temporal resolution, low latency and high dynamic range. However, state-of-the-art event- based algorithms either require too much computation time or have poor accuracy performance. In this paper, we propose an asynchronous real-time corner extraction and tracking algorithm for an event camera. Our primary motivation focuses on enhancing the accuracy of corner detection and tracking while ensuring computational efficiency. Firstly, according to the polarities of the events, a simple yet effective filter is applied to construct two restrictive Surface of Active Events (SAEs), named as RSAE+ and RSAE−, which can accurately represent high contrast patterns; meanwhile it filters noises and redundant events. Afterwards, a new coarse-to-fine corner extractor is proposed to extract corner events efficiently and accurately. Finally, a space, time and velocity direction constrained data association method is presented to realize corner event tracking, and we associate a new arriving corner event with the latest active corner that satisfies the velocity direction constraint in its neighborhood. The experiments are run on a standard event camera dataset, and the experimental results indicate that our method achieves excellent corner detection and tracking performance. Moreover, the proposed method can process more than 4.5 million events per second, showing promising potential in real-time computer vision applications.

## 1. Introduction

In recent years, owing to the development of computer vision technology and the enhancement of computer information processing capability, conventional frame-based cameras show important application value in several fields such as unmanned robot system, intelligent security and virtual reality. While widely adopted, frame-based cameras are not always optimal in many circumstances. For instance, when up against high velocity motions (easily causing blur) or high dynamic range scenes, frame-based cameras can hardly show robust performance. Furthermore, in a time period without motion, the images captured by frame-based cameras contain the same redundant information, which leads to a huge waste of computing resources.

As a new type of vision sensor, event cameras [1,2] are inspired by biology and operate in a very different way from frame-based cameras. Instead of capturing images at a fixed rate, event cameras respond to pixel brightness changes, called “events”, as they occur and output the time, locations and signs of the events asynchronously. Compared with frame-based cameras, event cameras show outstanding properties: very high temporal resolution (not suffering from motion blur) and low latency (both in the order of microseconds), very high dynamic range (140 dB vs. 60 dB of frame-based cameras) and low power consumption. In addition, event cameras only consider the information from “events”, thereby eliminating information redundancy and reducing computation. Event cameras show great potentials to overcome the challenges of high speed and dynamic range faced by conventional frame-based cameras, which have great application value in the field of virtual reality and robotics.

Since there are numerous advantages, several recent works [3,4,5,6,7,8,9,10,11,12,13,14] focus on processing the unconventional output of event cameras and unlocking their potentials. In event-based vision, the corner is one of the most fundamental features, and corner event tracking is usually used in many applications, such as target tracking [15,16], 3D Reconstruction [17,18] and motion estimation [19,20]. In 2015, the first corner event detection and tracking algorithm was proposed in Clady et al. [21]. In this paper, a nonlinear optimization method is used to fit planes from the Surface of Active Events (SAE), and corner events are labeled as the intersection of several planes. However, as the event signal contains much noise, the performance of plane fitting is less than satisfactory. Inspired by conventional frame-based Harris corner detector [22], Vasco et al. proposed an event-based Harris corner detector called “eHarris” [23]. In this method, event streams are used to construct an artificially binary frame, and they detect the Harris corners directly on this binary frame. Although this method provides satisfactory results, it has the poor computational efficiency due to using convolutions. In Mueggler et al. [24], an event-based Features from Accelerated Segment Test (FAST) corner detector, called “eFAST”, was proposed, which is inspired by the original FAST [25] and detects the corner events on SAE using only comparison operations. Because of its simple design principle, this method has high computational efficiency, but the performance of corner detection is not as effective as that in Vasco et al. [23]. Alzugaray and Chli proposed an event-based corner detection and tracking algorithm [26]. The corner detection algorithm is inspired by eFAST but it is more accurate and efficient than eFAST. However, this detector has limited changes in fundamental principle, the detection performance is still not comparable to that of eHarris. Additionally, the corner tracking algorithm associates the current corner with the latest active corner in its neighborhood, and constructs a tree-like structure to represent the trajectory for the same corner in spatio-temporal space. However, the tracking performance is not ideal due to lack of effective restrictions. In order to improve the tracking accuracy, Alzugaray and Chli processed events individually as they generated and proposed an asynchronous patch-feature tracker [27], but because of the huge computing burden, this method runs about 30× slower than the tracker in [26], and it has poor real-time computing capability. Li et al. proposed an event-based corner detection algorithm called FA-Harris [28]; in this algorithm, corner candidates are first selected by an improved eFAST and then refined by an improved eHarris detector. Although the accuracy has been effectively improved, this algorithm consumes a large amount of computation time and hardly runs in real-time due to the tedious eHarris-based method.

**Figure 1:**
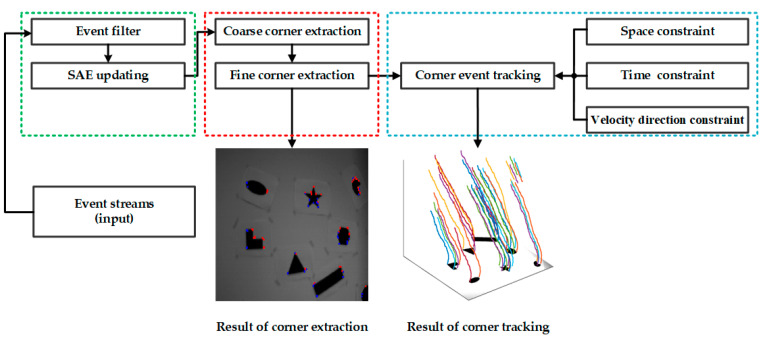
System overview of our proposed algorithm. The proposed algorithm consists of three units: a Surface of Active Events (SAEs) filter unit (green part), a coarse-to-fine corner extractor unit (red part) and a corner tracker unit (blue part).

Driven by the requirement for effective corner event processing, in this paper, we propose an asynchronous corner extraction and tracking algorithm for the event camera. Our primary motivation focuses on enhancing the repeatability of corner detection and the accuracy of corner tracking while ensuring computational efficiency. As shown in Figure 1, the proposed method consists of three units: an SAE filter unit (green part in Figure 1), a coarse-to-fine corner extractor unit (red part in Figure 1) and a corner tracker unit (blue part in Figure 1). The main contributions of this work are the following:

- Corner events are extracted by a new coarse-to-fine corner extractor. The coarse extractor with high calculation efficiency is used to extract corner candidates, and the fine extractor-based on a box filter only processes corner candidates. It significantly decreases the calculating amount and improves the efficiency without reducing the accuracy.
- A space, time and velocity direction constrained data association method is presented to realize corner event tracking, and we associate a new arriving corner event with the latest active corner that satisfies the velocity direction constraint in its neighborhood.

The remainder of this paper is organized as follows. The proposed method is detailed in Section 2. In Section 3, we perform our method on a publicly available event camera dataset and present the experimental results. Finally, we draw some conclusions and shed light on future work in Section 4.

## 2. Materials and Methods

In this paper, we propose an asynchronous corner extraction and tracking algorithm for an event camera. The main work of this paper has the following. Firstly, a simple yet effective filter is applied to filter out noise and redundant events. Afterwards, we extract corner events by a new coarse-to-fine corner extractor. Finally, a space, time and velocity direction constrained data association is presented to realize corner events tracking. The proposed method is detailed in the following subsections.

### 2.1. Filter of SAE

Event cameras operate in very differently from frame-based cameras; they only respond to pixel brightness changes, called “events”, and output the time, locations and signs of the events asynchronously. As an implementation of the send-on-delta transmission scheme [29], an event ***e*** = (*x*, *y*, *t*, *pol*) is generated at the pixel location (*x*, *y*) at time *t* if the absolute difference of logarithmic brightness *I*(*x*, *y*, *t*) reaches a threshold *K*, Formally

**Figure 2:**
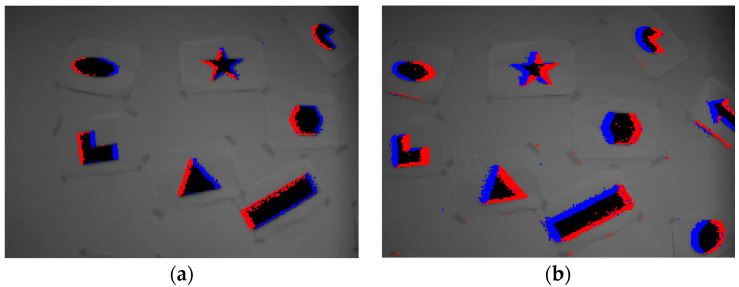
Events generated by two different motions: (**a**) the camera moves to the right, (**b**) the camera moves to the left. The blue and red dots represent events with positive and negative polarities, respectively.

**Figure 3:**
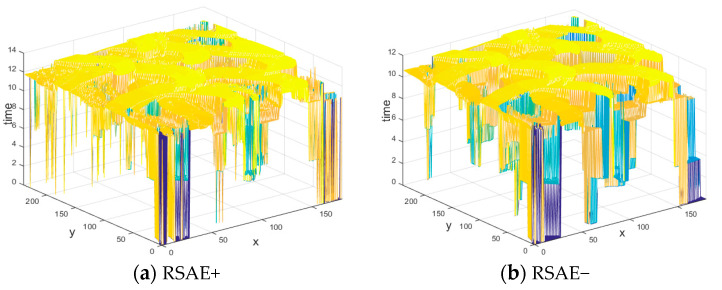
Two global Restrictive Surface of Active Events (RSAEs) constructed according to the polarities of the events: (**a**) RSAE+ constructed by the timestamp of the most recent positive event at each pixel, (**b**) RSAE− constructed by the most recent negative event at each pixel.

(1) I(x,y,t)-I(x,y,t-Δt)=pol⋅K

where *t*−∆*t* denotes the time when the last event was generated at the pixel location (*x*, *y*), *pol* denotes the polarity of the event (the sign of the logarithmic brightness change that is either 1 or −1). Normally, when an event is generated, it will be appended asynchronously to the event streams. Since there is no concept of image frames for event cameras, a notion of SAE is proposed in [30], which is used to store the timestamp of the most recent event at each pixel. When an event ***e*** = (*x*, *y*, *t*, *pol*) arrives, the value of SAE at (*x*, *y*) is updated as *SAE*(*x*, *y*)←*t*. Because of noises and hardware limitations, a sudden brightness change will generate several events at the same pixel almost instantly, so the latest timestamps stored in the SAE are not the exact time when the stimulus signals are generated. This will reduce the accuracy of corner event extraction and tracking. In order to solve this problem, a simple yet effective filter [26] is applied to construct a more restrictive SAE, named as RSAE. In the RSAE, events generated within a small time window *k* (typically 50 ms) at the same pixel will be ignored and not be used for updates. More precisely, when an event ***e*** = (*x*, *y*, *t*, *pol*) arrives, if *t-SAE(x*, *y*) > *k*, or if the polarity of the latest event at (*x*, *y*) differs from ***e***, the value of RSAE at (*x*, *y*) is updated as *RSAE*(*x*, *y*)←*t*. Owing to the special operating principle, in the same scene, different camera motions might generate different event streams. As shown in Figure 2, the brightness of the edge pixels will change with a moving camera, and different moving directions might generate different events, especially reflecting in the polarities of events. For this reason, according to the polarities of the events, we construct two more precise RSAEs, named as RSAE+ (see Figure 3a) and RSAE− (see Figure 3b) to replace the original RSAE. The above operations have the following two main benefits. Firstly, high contrast patterns (that is, edges) can be accurately represented in both time and space when brightness changes. Secondly, the noises and redundant events can be effectively filtered, which saves considerable calculation time.

### 2.2. Corner Event Extraction

Among the existing corner event extractors, eHarris [23] provides satisfactory results but has poor computational efficiency due to the use of convolutions. Conversely, eFAST [24] has high computational efficiency, but the performance is not as effective as eHarris. FA-Harris [28] adopts a coarse-to-fine extraction strategy; in this algorithm, corner candidates are first selected by an improved eFAST detector and then refined by an improved eHarris detector. Although the accuracy has been effectively improved, this algorithm consumes a large amount of computation time and hardly runs in real-time due to the tedious eHarris-based method. For this reason, in this paper, a new coarse-to-fine corner event extraction method is proposed. We first adopt Arc* [26] to extract corner candidates from event streams and then develop a box filter-based extractor to refine the corner candidates. Compared with the FA-Harris detector, our method significantly improves the efficiency without reducing the accuracy.

#### 2.2.1. Coarse Corner Extraction

**Figure 4:**
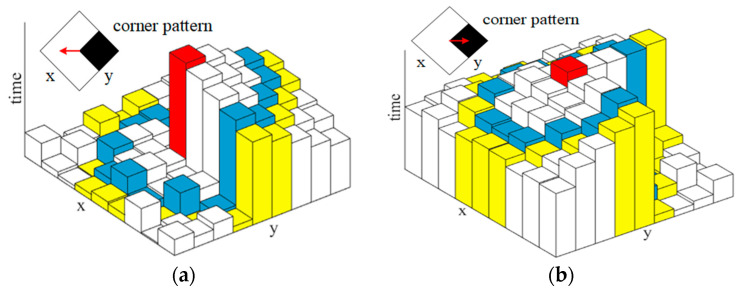
Illustrative examples of two entirely different local RSAEs generated by the same corner pattern with two different moving directions: (**a**) the corner pattern moves to the left, (**b**) the corner pattern moves to the right. The local RSAE is constructed by the 9 × 9 pixel-sized patch around ***e*** (the red element).

**Figure 5:**
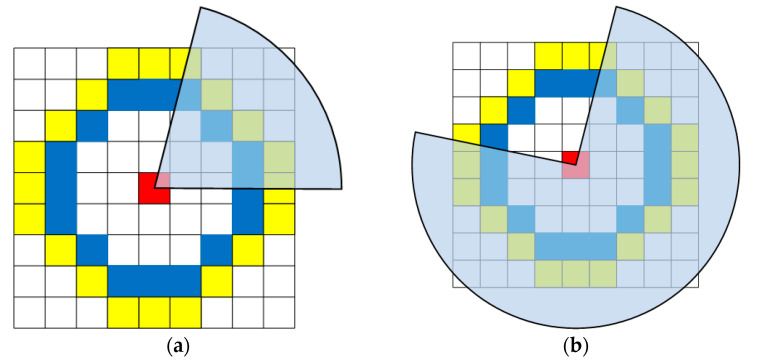
Schematic diagrams of coarse corner extraction in the two dimensional plane: (**a**) corresponding to the case in Figure 4a and (**b**) corresponding to the case in Figure 4b.

When an event ***e*** = (*x*, *y*, *t*, *pol*) arrives, we first update RSAE* (RSAE+ or RSAE−) according to the polarity of ***e***, by using the method in Section 2.1. Then Arc* [26] is adapted to extract corner candidates on the corresponding RSAE*. As shown in Figure 4, a moving corner pattern can generate a local RSAE* with two markedly distinct regions, and two different moving directions will create entirely different local RSAEs for the same corner pattern. Therefore, the corner candidates are extracted by searching for a continuous region of the local RSAE* with higher value than all other elements. More specifically, a 9 × 9 pixel-sized patch around ***e*** is selected on the RSAE*. For convenience, we only consider the pixels on two centered concentric circles with radius 3 and 4. For each circle, we search for a continuous arc with higher timestamps than all other pixels on the circle. On the inner circle (blue), the arc length *l_inner_* should be within the interval of [3,6], and on the outer circle (yellow), the arc length *l_outer_* should be within the interval of [4,8] (see Figure 5a). Alternatively, the arc length on the inner and outer circle should be within the interval of [10,13] and [12,16], respectively (see Figure 5b). In either case, if such an arc can be searched on both circles, the event is considered to be a corner candidate.

#### 2.2.2. Fine Corner Extraction

The method in Section 2.2.1 can efficiently extract corner candidates and remove invalid events. In order to further enhance the accuracy, a box filter-based method is developed to refine the corner candidates. For a corner candidate, the corresponding local RSAE* is used to construct a 9 × 9 pixel-sized local binary patch *T*. We search for the largest *n* numbers (the latest *n* events) in the local RSAE* and set the corresponding elements in patch *T* to 1; meanwhile, the rest of the elements in patch *T* are set to 0. As shown in Figure 5, the total number of elements in the 9 × 9 pixel-sized local patch is 81, and the total number of elements on the inner circle (blue) is 16. Therefore, in our work we set

(2) $$n=\mathrm{round}(l_{inner}\times 81/16)$$

where *l_inner_* is the arc length on the inner circle, which we have detailed in Section 2.2.1 and round(∙) denotes the operation of rounding to the nearest integer. Similar to eHarris [23], we construct a 2 × 2 sized Hessian matrix ***H*** as follows:

(3) $$H=\left[\begin{array}{l} A\;B\\ B\;C \end{array}\right]$$

where

(4) $$A=\sum_{x,y}w(x,y)\cdot T_{x}^{2}(x,y)=L_{xx}(x,y)⊗T(x,y)$$

(5) $$B=\sum_{x,y}w(x,y)\cdot T_{x}(x,y)T_{y}(x,y)=L_{xy}(x,y)⊗T(x,y)$$

(6) $$C=\sum_{x,y}w(x,y)\cdot T_{y}^{2}(x,y)=L_{yy}(x,y)⊗T(x,y)$$

where T(x,y) is the element value of the patch *T* at the pixel location (*x*, *y*), $T_{x}(x,y)$ and $T_{y}(x,y)$ are the gradients of T(x,y) in the *x* and *y* directions, respectively, w(x,y) is a Gaussian convolution kernel with a standard deviation of 1.2. $L_{xx}(x,y)$,$L_{yy}(x,y)$ and $L_{xy}(x,y)$ are Gaussian second-order differential templates in the *x-x*, *y-y* and *x-y* directions, respectively, and ⊗ denotes the operation of convolution operation. For each corner candidate, a score *R* can be calculated as

(7) $$R=\det(H)=\;AC-B^{2}$$

then we compare the score *R* with a threshold to determine whether this candidate is a corner event.

**Figure 6:**
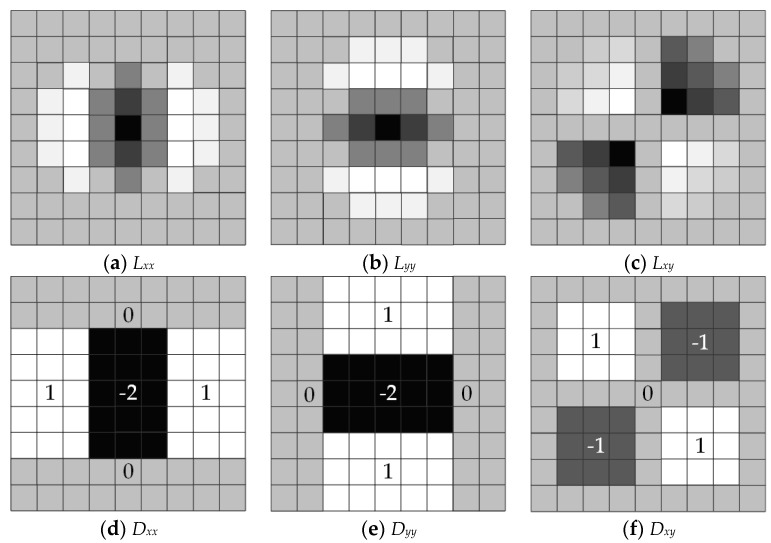
The 9 × 9 pixel-sized Gaussian second-order differential templates and the corresponding box filter templates. (**a**–**c**) are Gaussian second-order differential templates in the *x-x*, *y-y* and *x-y* directions, respectively, and (**d**−**f**) are the box filter templates corresponding to (**a**−**c**), respectively. In these templates, the elements are within the interval of [–2, 1], and dark colors correspond to small values, whereas light colors correspond to large values. The elements in Gaussian second-order differential templates have different values; for the sake of convenience, we have not labeled the exact values. The box filter templates are only composed of several rectangular regions, and each rectangular region is filled with the same value. We have labeled the exact value for each rectangular region.

In practical applications, it is a complicated and time-consuming process to calculate the Hessian Matrix ***H*** through the above formulas. In order to improve the efficiency of the algorithm, we introduce box filter templates [31] to approximate Gaussian second-order differential templates. The box filter templates and the corresponding Gaussian second-order differential templates are shown in Figure 6. The elements in Gaussian second-order differential templates have different values, however, the box filter templates are only composed of several rectangular regions, and each rectangular region is filled with the same value. Therefore, elements in the Hessian matrix ***H*** can be approximated as

(8) $$A\approx D_{xx}(x,y)⊗T(x,y)$$

(9) $$B\approx D_{xy}(x,y)⊗T(x,y)$$

(10) $$C\approx D_{yy}(x,y)⊗T(x,y)$$

where $D_{xx}(x,y)$,$D_{xy}(x,y)$ and $D_{yy}(x,y)$ are the box filter templates corresponding to the templates in Figure 6d,e,f, respectively. The box filter templates can transform a convolution operation into the simple addition of values between different rectangular regions. Compared with the FA-Harris detector, our method greatly reduces the computation amount and improves computational efficiency.

### 2.3. Corner Event Tracking

When corner events are extracted asynchronously, how to establish data associations between these corners is a big challenge. Alzugaray and Chli [26] associate the current corner with the latest active corner in its neighborhood and construct a tree-like structure to represent the trajectory for the same corner in spatio-temporal space. However, the tracking performance of this method is not ideal due to lack of effective restrictions. In order to enhance the tracking accuracy, we improve on Alzugaray’s method and propose a space, time and velocity direction constrained corner event tracking method in this paper.

**Figure 7:**
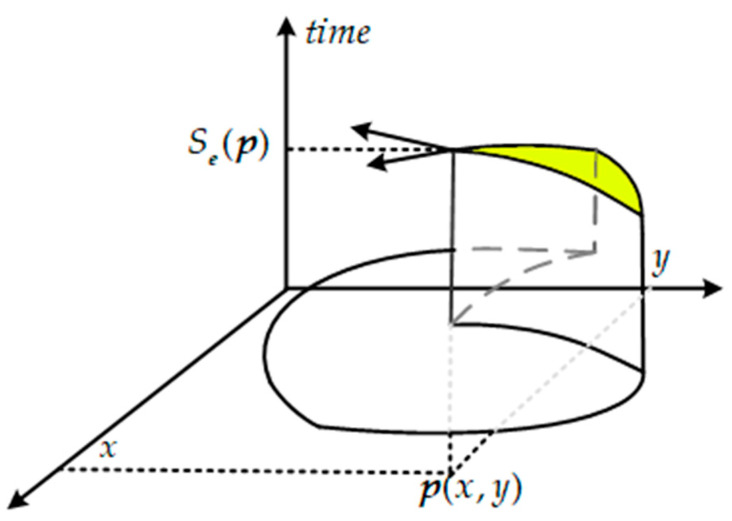
Schematic diagram of the velocity direction computation. The velocity vector is equal to the inverse of the first partial derivative of RSAE.

When the above event ***e*** = (*x*, *y*, *t*, *pol*) is determined as a corner, as shown in Figure 7, we define the corresponding local RSAE* as a function *S**_e_***(∙) which projects the position ***p***(*x*, *y*) to time *t*

(11) $$S_{e}(p)=t$$

where time *t* is an increasing quantity. The first partial derivative of *S**_e_***(∙) can be written as

(12) $$\frac{\partial S_{e}}{\partial x}(x,y_{0})=\frac{dS_{e}|{}_{y_{0}}}{dx}(x)=\frac{1}{v_{x}(x,y_{0})}$$

(13) $$\frac{\partial S_{e}}{\partial y}(x_{0},y)=\frac{dS_{e}|{}_{x_{0}}}{dy}(y)=\frac{1}{v_{y}(x_{0},y)}$$

where $S_{e}|{}_{x_{0}}$ and $S_{e}|{}_{y_{0}}$ denote *S**_e_***(∙) restricted to *x* = *x*_0_ and *y* = *y*_0,_ respectively. The gradient of *S**_e_***(∙) can be written as

(14) $$\nabla S_{e}={\left(\frac{1}{v_{x}},\frac{1}{v_{y}}\right)}^{T}$$

the gradient vector $\nabla S_{e}$ measures the change of time versus position, and it is also the inverse of the velocity vector. In order to estimate the gradient vector $\nabla S_{e}$ robustly, we assume that the local velocity vector is constant over a very small time on the local RSAE*. This is also equal to the assumption that the elements in the local RSAE* with higher timestamps (yellow in Figure 7) is a local plane, because the first partial derivative of *S**_e_***(∙) is the inverse of the velocity vector, and a constant local velocity vector produces a constant change in *S**_e_***(∙). Assuming the corner event ***e*** = (*x*, *y*, *t*, *pol*) belongs to a local plane with parameters $N={\left(a,b,c,d\right)}^{T}$, it satisfies

(15) $$(a\;b\;c\;d)\left(\begin{array}{l} x\\ y\\ t\\ 1 \end{array}\right)=0$$

In addition, the elements in the local RSAE* with higher timestamps also satisfy Equation (15), and the parameters $N={\left(a,b,c,d\right)}^{T}$ can be solved by a nonlinear least squares problem as

(16) $$\underset{N}{\arg\min}\sum_{i}{\left|N^{T}\left(\begin{array}{l} x_{i}\\ y_{i}\\ t_{i}\\ 1 \end{array}\right)\right|}^{2}$$

then the velocity vector can be calculated by

(17) $$v^{T}={\left(v_{x},v_{y}\right)}^{T}={\left(-\frac{c}{a},-\frac{c}{b}\right)}^{T}$$

**Figure 8:**
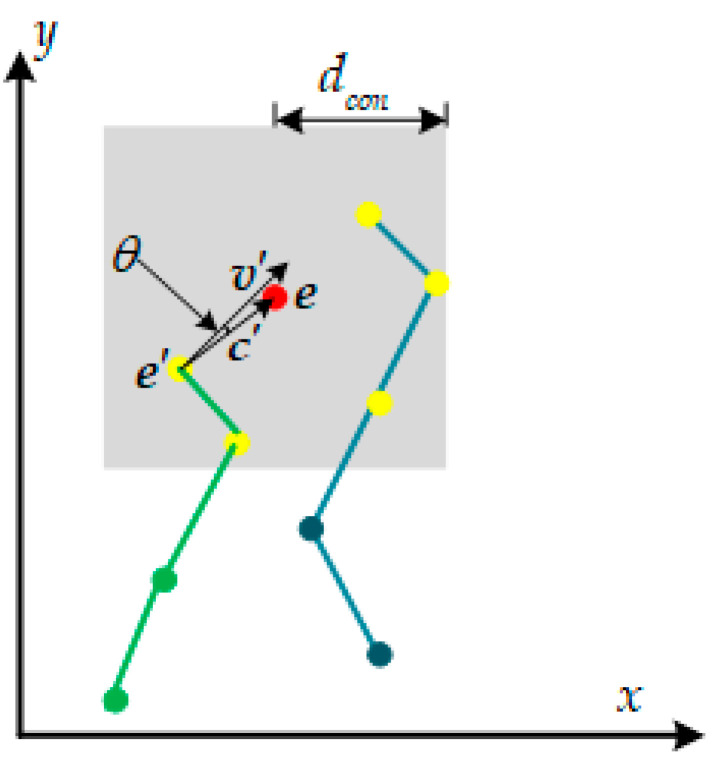
Schematic diagram of the proposed data association method. For a new arriving corner event ***e*** (red dot), we firstly search for the corners that satisfy the time and space constraints (yellow dots) and then search for the corner associated with the latest active corner that satisfies the velocity direction constraint. Green lines and blue lines indicate trajectories of two different corners.

As shown in Figure 8, we associate a new corner event ***e*** = (*x*, *y*, *t*, *pol*) with the latest active corner that satisfies the velocity direction constraint in its neighborhood. The neighborhood is defined as a region with a maximum range of *d_con_* pixels centered around ***e***; in our work we set *d_con_* = 5. We also introduce a time constraint, so that corners more than *t_con_* seconds later than ***e*** in the neighborhood will not be considered, which can effectively prevent data association with older corners. In our work we set *t_con_* = 0.1. In addition to the time and space constraints, we add a velocity direction constraint for accurate tracking. More precisely, for a new arriving corner event ***e***, we firstly search for the corners that satisfy the above time and space constraints in its neighborhood, then sort them in chronological order and store them in a container *C_neigh_*. For each corner event e’=(x’,y’,t’,pol’) in *C_neigh_*, the connection vector ***c***’ between ***e*** and ***e***’ can be calculated as $c’={\left(x-x’,y-y’\right)}^{T}$. Suppose we denote the velocity vector of ***e***’ as ***v***’, the angle between ***v***’ with ***c***’ can be calculated as

(18) $$\theta =\arccos\frac{v’\cdot c’}{\left|v’\right|\left|c’\right|}$$

in our work, ***e***’ is considered to satisfy the velocity vector constraint if θ<5 degrees. Then we associate ***e*** with the latest active corner event that satisfies the above constraint in *C_neigh_* and realize an accurate tracking of corner events.

## 3. Results

The proposed algorithm is performed on the publicly available event camera dataset [32]. This dataset is recorded by a DAVIS240C, which captures both events and intensity images with the resolution of 240 × 180 pixels. Our algorithm only processes the event streams, and the intensity images are adopted to collect ground truth. Because of space limitations, we select four representative scenes (shapes, dynamic, poster and boxes) with increasing complexity and event rate. Note that in order to ensure the fairness of the experiments, the selected scenes used in our experiments are same as those in Alzugaray and Chli [26]. We compare our algorithm with other advanced algorithms in term of extraction and tracking accuracy, and computational efficiency. In addition, the experimental results are analyzed. The following section details the ground truth collection method and experiment results.

### 3.1. Ground Truth

**Figure 9:**
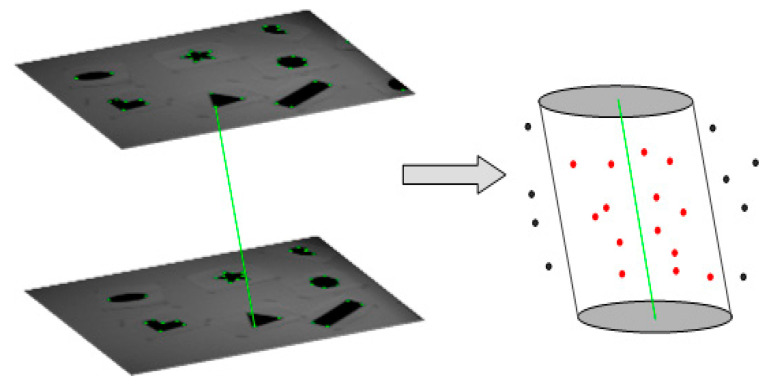
Schematic diagram of the ground truth collection method. The green corners are extracted by the original Harris method and tracked by KLT on intensity images. The solid green line is the trajectory of one of the corners. The 5 pixel-sized neighborhood of the trajectory is enlarged and represented as an oblique cylinder. In our metrics, we only concern the events within 5 pixel-sized neighborhood of the KLT-based tracking (events in the oblique cylinder).

Since there is no data directly reflecting the actual behavior of the DAVIS240C in this dataset, how to collect ground truth for evaluating the performance is one of the difficulties in our work. In references [21,23], corner events are manually determined and labelled in event streams, however, these methods are only applicable to clear corners in simple scenes due to tedious manual work. In Mueggler et al. [24], an automatic determining and labelling method is proposed to calculate the spatiotemporal coordinates of the corner events, however, this method suffers from tremendous missed detections. Similar to the method in Alzugaray and Chli [26], the available intensity images are used to collect ground truth in this paper, and we use original Harris [22] to extract corners and track the corners by KLT [33]. In order to match the temporal resolution of the corner events, we adopt a cubic splines method to interpolate corresponding coordinates in the image plane. In our accuracy metrics, we only concern the events which are not filtered in Section 2.1. As shown in Figure 9, corners only generated within 5 pixel-sized neighborhood of the KLT-based tracking are concerned in our metrics. Since the selected dataset is recorded with increasing velocity, we just select the data of the first 10 s, which can reduce the influence on ground truth caused by high speed motion blur.

### 3.2. Experimental Evaluation

#### 3.2.1. Corner Extraction Performance

**Figure 10:**
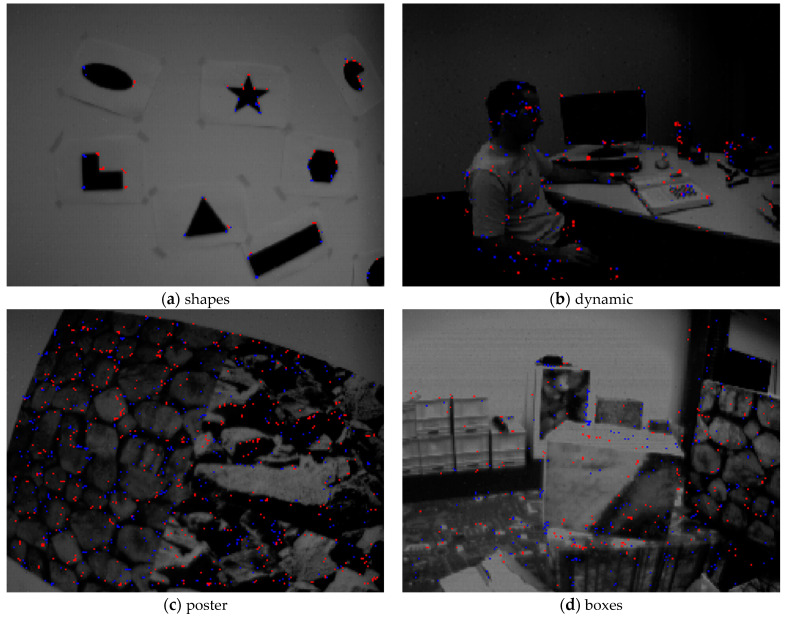
Illustrative examples of corner event extraction by our method in four different scenes. We synthesize all corner events within 100 milliseconds and display on an intensity image. Red and blue dots represent negative and positive polarities of the corner events, respectively.

Illustrative examples of corner event extraction by our method are shown in Figure 10. For each scene, we synthesize all corner events within 100 milliseconds and display on an intensity image. In Figure 10, different colors represent different polarities of the corner events; red and blue represent negative and positive respectively. Similar to [26], we adopt True Positive Rate (TPR) and False Positive Rate (FPR), a standard framework of binary classification, to evaluate the performance of the different extractors. A corner event is marked as True Positive (TP) if it is within 3.5 pixels of the KLT-based tracking, or False Positive (FP) if it is within the interval of [3.5, 5] pixels. Conversely, an event which is not considered as a corner but is within 3.5 pixels of the KLT-based tracking is marked as False Negative (FN), or True Negative (TN) if it is within the interval of [3.5, 5] pixels. TPR is calculated as TPR = TP/(TP + FN), and FPR is calculated as FPR = FP/(FP + TN). The TPR and FPR of different corner event extractors for different scenes are shown in Table 1 and Table 2, respectively. Table 3 reports the Corner Event Rate (CER) of different extractors in four different scenes. CER is defined as the total number of corner events versus the total number of events.

The statistical results show that in a simple scene (shapes) all five methods have higher CER, TPR and FPR scores than that in complex scenes (dynamic, poster and boxes). It is because that there are more events in complex scenes than that in simple scene, and numerous events are considered as non-corner events in complex scenes. It can be seen from Table 3 that Arc* has higher CER scores than other extractors, that is to say, Arc* considers more events as corners. Additionally, Arc* has lower TPR and higher FPR scores, so it can be inferred that Arc* slightly enhances the CER at great sacrifice of accuracy; therefore, the corner events extracted by Arc* contain more incorrect detections. eFAST has the lowest CER scores because it only considers the condition in Figure 5a and ignores the condition in Figure 5b; therefore, many corners are not detected by eFAST. Owing to the coarse-to-fine corner extraction strategy, the CER scores of our method are comparable to the FA-Harris method but are slightly lower than the eHarris method; note that our method achieves results about 20× faster than eHarris. As shown in Table 1 and Table 2 that the TPR scores of our method are comparable to eHarris and FA-Harris, and they are obviously better than the other two algorithms. Moreover, our method and the FA-Harris method have lower FPR scores than the other three extractors. That is to say, our method and the FA-Harris method have better precision performance then the above five extractors, which is largely due to the coarse-to-fine corner extraction strategy. Because we develop a box filter-based method to replace the tedious eHarris-based method in fine corner extraction process, our method achieves results about 3× faster than the FA-Harris method. Compared with FA-Harris, our method significantly decreases the calculating amount and improves the efficiency without reducing the accuracy.

#### 3.2.2. Corner Tracking Performance

**Figure 11:**
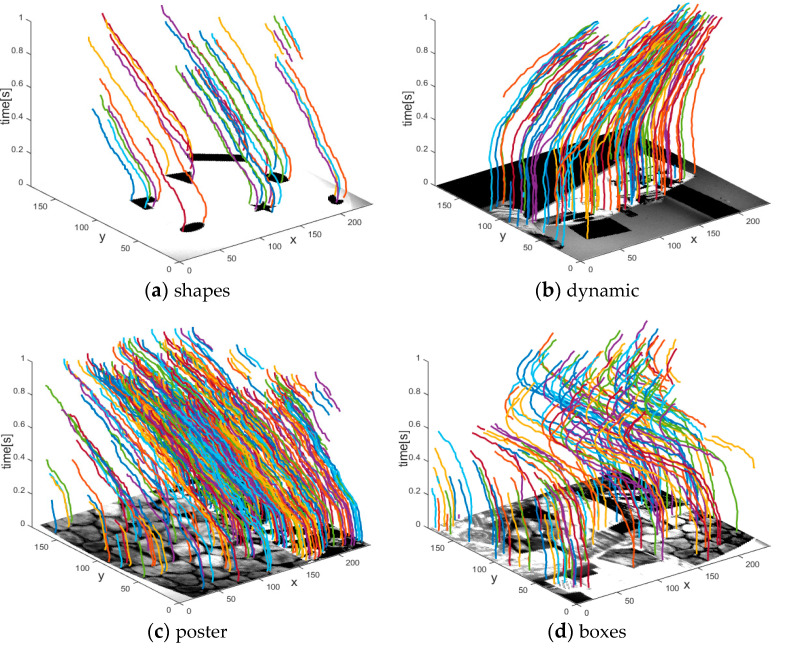
Illustrative examples of corner event tracking by our method in four different scenes. Date associations for different corner events are marked as solid lines in different colors. We synthesize the trajectories on the corresponding intensity image for viewing convenience.

Illustrative examples of corner event tracking by our method are shown in Figure 11. Similar to [26], we adopt Mean Absolute Error (MAE), Valid Track Rate (VTR) and Mean Track Lifetime (MTL) to evaluate the performance and summarize the results in Table 4. MAE is defined as the mean absolute distance between event-based tracking and KLT- based tracking. We consider event-based tracking as valid tracking if the MAE is within 5 pixels, otherwise we consider it as invalid tracking. For each scene, VTR is defined as the total number of valid tracking versus the total number of corner event tracking. Our tracking algorithm associates a new arriving corner event with the latest active corner that satisfies the velocity direction constraint in its neighborhood, so it is based on the assumption that the corner events are continuously and steadily detected. If the assumption is broken, the new arriving corner event will be considered as a new corner or even generate an incorrect data association. MTL is defined as the mean duration for the corner event tracking which matches the same intensity-based tracking validly. As shown in Table 4, in four different scenes, our method achieves less MAE and higher VTR than the algorithm in [26]. This is because our method adds a velocity direction constraint on Alzugaray’s method, which can effectively eliminate incorrect data associations. Owing to this strict constraint, the MTL of our method is slightly less than that in [26]. Note that our method effectively enhances the tracking accuracy at slight sacrifice of MTL.

#### 3.2.3. Computational Performance

All the above algorithms are implemented by single threaded C++ programs and run on an Intel(R) Core(TM) i7 CPU with 2.80 GHz and 16 GB of RAM. Table 5 presents the average time consumption of a single event and the maximum processing rate in millions of events per second (Mev/s) for each algorithm. Intuitively, our method has a good performance in computational consumption. On average, our proposed extractor achieves results about 20× faster than the eHarris method, but about 1.5× slower than Arc*. This improvement is because we propose a coarse-to-fine corner extraction method; the coarse extraction with high calculation efficiency is used to extract corner candidates, and the fine extraction only processes corner candidates, not all events. Although our extractor is more computationally expensive than Arc*, the accuracy performance is significantly better. Our proposed extractor also achieves results about 3× faster than the FA-Harris method, because we develop a box filter-based method to replace the tedious eHarris-based method in the fine corner extraction process. Compared with FA-Harris, our method significantly decreases the calculating amount and improves the efficiency without reducing the accuracy. Table 5 also reports the average time consumption of corner event tracking. Superficially, the proposed tracking algorithm takes much more time than extraction; note that only corner events, not all events, are concerned in the tracking process, in fact the time consumption of the tracking process accounts for less than 45% of the total computation time. Furthermore, a single corner tracking time of our method is slower than that in [26], but because of the fine extraction process, our algorithm extracts relatively less but more accurate corner events than Arc*, which also saves a lot of computing time in the whole tracking process. Therefore, our work has great potential in real-time computer vision applications.

## 4. Conclusions

We propose an asynchronous real-time corner extraction and tracking algorithm for event cameras and show its excellent accuracy performance and good computational efficiency. In our algorithm, corner events are asynchronously extracted by a coarse-to-fine extractor and associated with the latest active corners that satisfy the velocity direction constraints in their neighborhood. Compared with the method in [26], our method effectively enhances the accuracy at slight sacrifice of computational efficiency. Experiment results also indicate that the proposed method can process more than 4.5 million events per second, showing great potential in real-time computer vision applications. Our further interest lies in applying our work to SLAM in challenging environments (high velocity movements or high dynamic range scenes) while existing frame-based SLAM algorithms have limitations.

## Figures and Tables

**Table 1:** The True Positive Rate (%) of different extractors in four different scenes. The best performance across each experiment and metric is highlighted in bold.

Scenes	Algorithm				
	eFAST [24]	Arc * [26]	eHarris [23]	FA-Harris [28]	Our Method
shapes	33.21	45.31	57.52	57.64	**57.83**
dynamic	21.74	25.70	**46.18**	45.85	45.97
poster	15.10	22.82	42.72	**43.31**	43.17
boxes	12.29	17.65	39.36	**40.03**	39.97

**Table 2:** The False Positive Rate (%) of different extractors in four different scenes. The best performance across each experiment and metric is highlighted in bold.

Scenes	Algorithm				
	eFAST [24]	Arc* [26]	eHarris [23]	FA-Harris [28]	Our Method
shapes	10.08	12.47	9.39	5.42	**5.21**
dynamic	5.57	7.61	5.43	**3.06**	3.19
poster	5.41	7.08	5.10	**1.87**	1.96
boxes	4.40	6.17	4.25	1.68	**1.54**

**Table 3:** The Corner Event Rate (%) of different extractors in four different scenes.

Scenes	Algorithm				
	eFAST [24]	Arc * [26]	eHarris [23]	FA-Harris [28]	Our Method
shapes	8.82	13.49	11.28	10.17	10.26
dynamic	5.91	8.65	7.36	6.29	6.16
poster	5.72	9.43	7.74	6.80	6.62
boxes	5.16	8.12	6.67	6.02	6.17

**Table 4:** The tracking performance evaluation of our method. The best performance across each experiment and metric is highlighted in bold.

Scenes	Algorithm	MAE (Pixels)	VTR (%)	MTL (s)
shapes	method in [26]	2.25	82.39	**1.13**
	our method	**1.57**	**83.11**	1.07
dynamic	method in [26]	2.64	60.21	**0.77**
	our method	**1.61**	**78.28**	0.71
poster	method in [26]	2.82	54.64	**0.64**
	our method	**1.51**	**74.43**	0.62
boxes	method in [26]	2.88	53.25	**0.67**
	our method	**1.74**	**75.72**	0.64

**Table 5:** The computational performance of different corner event extraction and tracking algorithms.

Algorithm		Time per Event (*μs*/event)	Max. Event Rate (Mev/s)
**Extraction method**	eHarris [23]	5.36	0.17
	eFAST [24]	0.16	6.21
	Arc * [26]	0.13	7.63
	FA-Harris [28]	0.68	1.49
	our method	0.22	4.52
**Tracking method**	method in [26]	2.14	0.44
	our method	3.98	0.25

## Data Availability

Publicly available datasets were analyzed in this study. This data can be found here: http://rpg.ifi.uzh.ch/davis_data.html.
